# Supplementary material for: Daratumumab Interferes with Allogeneic Crossmatch Impacting Immunological Assessment in Solid Organ Transplantation
Source: J Clin Med. 2022 Oct 14;11(20):6059. doi: 10.3390/jcm11206059 (PMC9605360; doi:10.3390/jcm11206059)
Supplement: Supplementary file 1 [file jcm-11-06059-s001.zip › Figure S1_Representative Luminex HLA Antibody Profile.pdf]

Class I

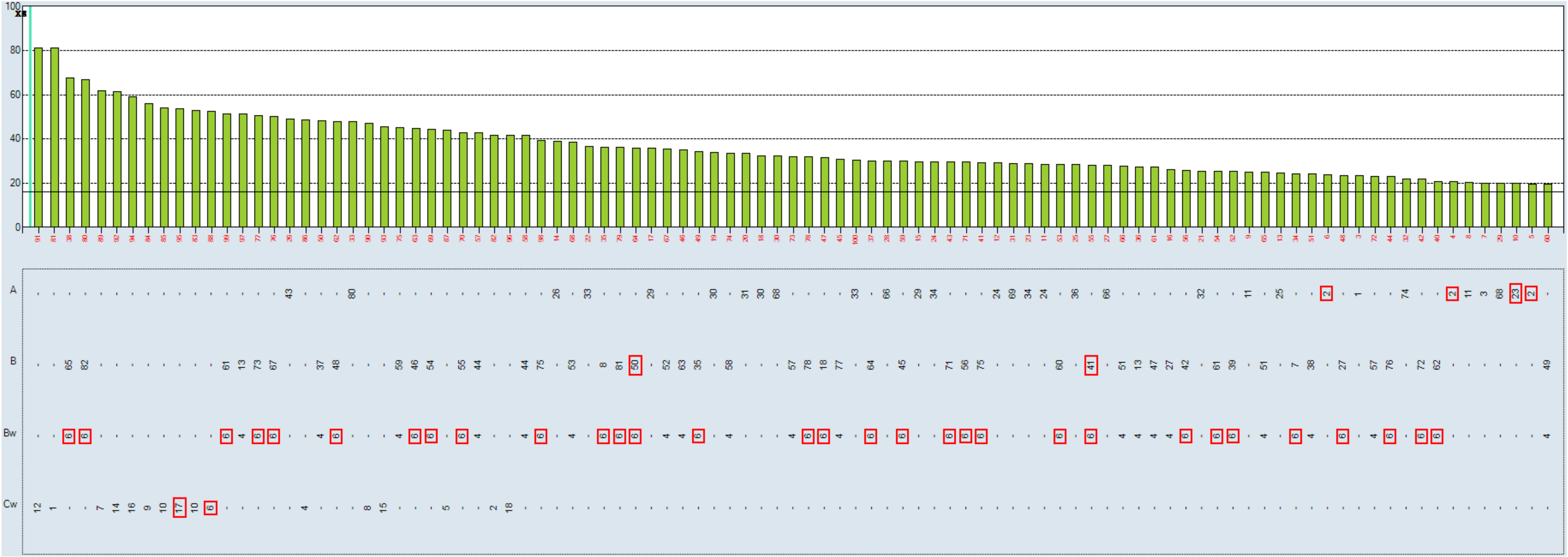

Class II

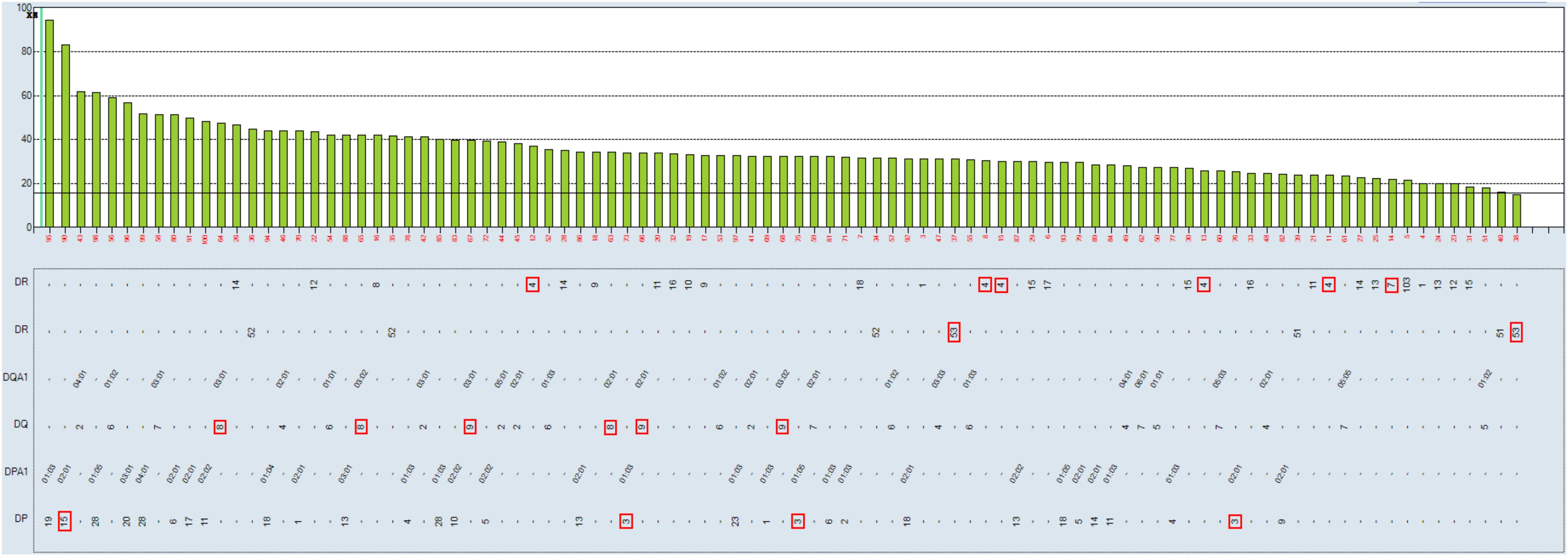

Figure S1. Representative Luminex single antigen bead results of the patient. Specificities in the red boxes are the patient's self-HLA antigens which are some of the highest-ranking beads. The serum was treated with EDTA to eliminate inhibition/prozone.
