# Supplementary material for: Daratumumab Interferes with Allogeneic Crossmatch Impacting Immunological Assessment in Solid Organ Transplantation
Source: J Clin Med. 2022 Oct 14;11(20):6059. doi: 10.3390/jcm11206059 (PMC9605360; doi:10.3390/jcm11206059)
Supplement: Supplementary file 1 [file jcm-11-06059-s001.zip › Table S1_Deceased Donor Crossmatch History.pdf]

Deceased Donor Crossmatch History

Deceased Donor		Crossmatch	Recipient Serum		T Cell Crossmatch*		B Cell Crossmatch*	
Number	Blood Type	Date	Category	Date	Result	MCS	Result	MCS
1	A1	06/27/2020	Current	06/19/2020	POSITIVE	61	POSITIVE	152
2	A1B	07/13/2020	Current	06/19/2020	Negative	39	POSITIVE	74
			Historic	06/06/2020	Negative	37	POSITIVE	84
3	A1	07/20/2020	Current	06/19/2020	Negative	21	POSITIVE	154
4	A1B	07/26/2020	Current	06/19/2020	POSITIVE	132	POSITIVE	197
5	Non-A1B	07/28/2020	Current	06/19/2020	POSITIVE	80	POSITIVE	219
6	A1	07/29/2020	Current	06/19/2020	POSITIVE	85	POSITIVE	139
7	A	08/01/2020	Current	06/19/2020	POSITIVE	94	POSITIVE	181
8	A	08/05/2020	Current	06/19/2020	POSITIVE	116	POSITIVE	215
9	A1B	08/08/2020	Current	08/03/2020	POSITIVE	128	POSITIVE	192
			Historic	06/19/2020	POSITIVE	133	POSITIVE	192
10	A	08/11/2020	Current	08/03/2020	POSITIVE	132	POSITIVE	215
			Historic	06/19/2020	POSITIVE	134	POSITIVE	216
11	A1	08/12/2020	Current	08/03/2020	Negative	25	POSITIVE	164
			Historic	06/19/2020	Negative	22	POSITIVE	164
12	A1	08/14/2020	Current	08/03/2020	POSITIVE	88	POSITIVE	219
			Historic	06/19/2020	POSITIVE	87	POSITIVE	217
13	A1	08/17/2020	Current	08/03/2020	POSITIVE	58	POSITIVE	168
			Historic	06/19/2020	POSITIVE	63	POSITIVE	172
14	A1	08/24/2020	Current	08/03/2020	Negative	10	Negative	28
			Historic	06/19/2020	Negative	10	Negative	28
15	A1	09/06/2020	Current	08/31/2020	POSITIVE	78	POSITIVE	174
			Historic	06/19/2020	Negative	33	POSITIVE	130
16	A1	09/09/2020	Current	08/31/2020	POSITIVE	72	POSITIVE	194
			Historic	06/19/2020	POSITIVE	70	POSITIVE	192
17	A1	09/12/2020	Current	08/31/2020	POSITIVE	60	POSITIVE	203
			Historic	06/19/2020	Negative	37	POSITIVE	162
18	A1	09/18/2020	Current	08/31/2020	Negative	36	POSITIVE	90
			Historic	06/19/2020	Negative	30	POSITIVE	88
19	A	09/19/2020	Current	08/31/2020	POSITIVE	69	POSITIVE	101
			Historic	06/19/2020	POSITIVE	73	POSITIVE	106
20	AB	10/06/2020	Current	09/28/2020	POSITIVE	57	POSITIVE	142
			Historic	06/19/2020	POSITIVE	55	POSITIVE	142
21	B	10/09/2020	Current	10/05/2020	Negative	26	POSITIVE	185
			Historic	06/19/2020	Negative	23	POSITIVE	186
22	A1	10/09/2020	Current	10/05/2020	POSITIVE	158	POSITIVE	233
			Historic	06/19/2020	POSITIVE	155	POSITIVE	239
23	A1	10/14/2020	Current	10/05/2020	POSITIVE	113	POSITIVE	141
			Historic	06/19/2020	POSITIVE	89	POSITIVE	142
24	A1	10/23/2020	Current	10/12/2020	POSITIVE	52	POSITIVE	137
25	A1	10/30/2020	Current	10/26/2020	POSITIVE	54	POSITIVE	109
26	A1	11/06/2020	Current	10/26/2020	POSITIVE	45	POSITIVE	129
			Historic	10/12/2020	POSITIVE	49	POSITIVE	137
27	AB	11/06/2020	Current	10/26/2020	POSITIVE	127	POSITIVE	221

MCS, Median Channel Shift
*Crossmatch positive cutoff: T cell > +39 MCS, B cell > +70 MCS
