# Supplementary material for: Daratumumab Interferes with Allogeneic Crossmatch Impacting Immunological Assessment in Solid Organ Transplantation
Source: J Clin Med. 2022 Oct 14;11(20):6059. doi: 10.3390/jcm11206059 (PMC9605360; doi:10.3390/jcm11206059)

Surrogate Donor	Lymphocyte Source	DTT treatment	T-cell CD38 Expression	B-cell CD38 Expression	T-cell HLA Class I Expression	B-cell HLA Class I Expression	B-cell HLA Class II Expression
A	Peripheral Blood	Untreated	253	262	501	464	536
		0.05M, 10 min at 37°C	230	179	535	474	535
		0.05M, 20 min at 37°C	243	183	540	498	541
		0.05M, 30 min at 37°C	242	183	546	494	535
		0.10M, 10 min at 37°C	240	180	539	499	548
		0.10M, 20 min at 37°C	242	180	545	494	539
		0.10M, 30 min at 37°C	243	185	531	486	533
B	Peripheral Blood	Untreated	257	255	469	522	639
		0.05M, 10 min at 37°C	246	217	510	555	645
		0.05M, 20 min at 37°C	246	215	522	562	646
		0.05M, 30 min at 37°C	246	218	520	556	642
		0.10M, 10 min at 37°C	247	214	507	546	645
		0.10M, 20 min at 37°C	248	213	512	552	637
		0.10M, 30 min at 37°C	249	219	514	542	635
C	Spleen	Untreated	275	283	464	488	674
		0.05M, 10 min at 37°C	233	231	520	501	677
		0.05M, 20 min at 37°C	234	228	523	494	670
		0.05M, 30 min at 37°C	240	225	528	490	647
		0.10M, 10 min at 37°C	231	225	518	489	666
		0.10M, 20 min at 37°C	235	223	525	492	663
		0.10M, 30 min at 37°C	239	233	523	479	654

Note: Values are expressed in Median Fluorescence Channels (MCF)

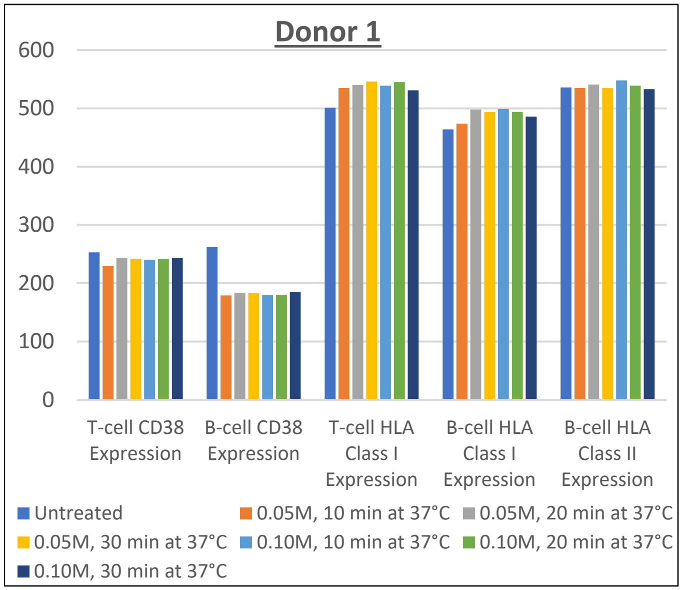

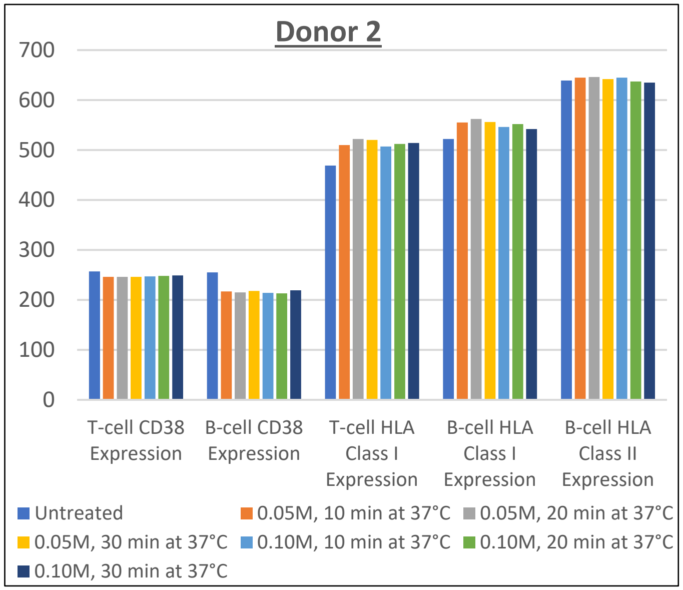

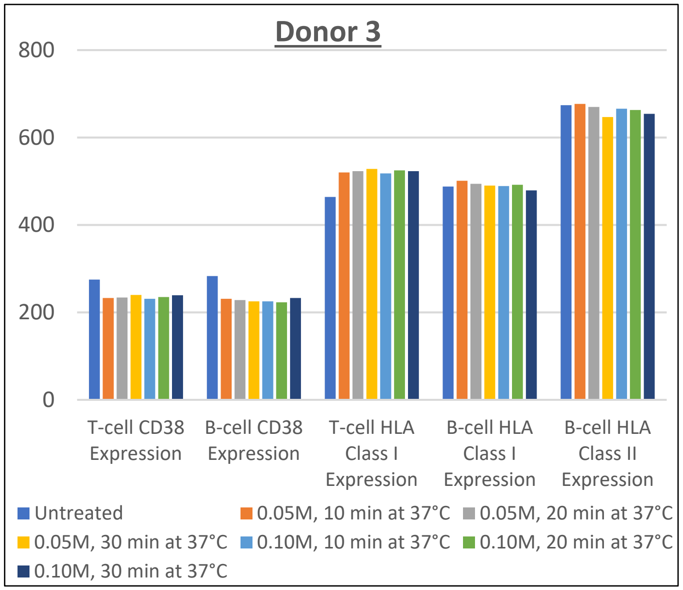

Supplement: Supplementary file 1 [file jcm-11-06059-s001.zip › Table S2_Impact of DTT Concentration and Incubation Time on CD38 Expression.pdf]
