# Supplementary material for: Daratumumab Interferes with Allogeneic Crossmatch Impacting Immunological Assessment in Solid Organ Transplantation
Source: J Clin Med. 2022 Oct 14;11(20):6059. doi: 10.3390/jcm11206059 (PMC9605360; doi:10.3390/jcm11206059)
Supplement: Supplementary file 1 [file jcm-11-06059-s001.zip › Table S3_Surrogate Crossmatch with DTT-Treated Cells and DTT-Treated Sera.pdf]

			Neat + Pronase				DTT Treatment 10 mins at 37°C + Pronase				DTT Treatment 30 mins at 37°C + Proanse			
			T-Cell		B-Cell		T-Cell		B-Cell		T-Cell		B-Cell	
	Serum Date	Serum Treatment	MCS	Result	MCS	Result	MCS	Result	MCS	Result	MCS	Result	MCS	Result
Surrogate Donor A	6/19/2021	Neat	42	POS	79	POS	-8	neg	-57	neg	-12	neg	-60	neg
	8/31/2021	Neat	42	POS	86	POS	-14	neg	-67	neg	-14	neg	-65	neg
	10/5/2021	Neat	42	POS	91	POS	-18	neg	-63	neg	-23	neg	-68	neg
	10/12/2021	Neat	41	POS	83	POS	-15	neg	-63	neg	-21	neg	-68	neg
	10/26/2021	Neat	42	POS	85	POS	-15	neg	-59	neg	-21	neg	-81	neg
	6/19/2021	DTT	45	POS	101	POS	NT	NT	NT	NT	NT	NT	NT	NT
	8/31/2021	DTT	42	POS	106	POS	NT	NT	NT	NT	NT	NT	NT	NT
	10/5/2021	DTT	47	POS	97	POS	NT	NT	NT	NT	NT	NT	NT	NT
	10/12/2021	DTT	44	POS	113	POS	NT	NT	NT	NT	NT	NT	NT	NT
	10/26/2021	DTT	46	POS	104	POS	NT	NT	NT	NT	NT	NT	NT	NT
Surrogate Donor B	6/19/2021	Neat	23	neg	127	POS	-11	neg	-67	neg	-14	neg	-62	neg
	8/31/2021	Neat	21	neg	126	POS	-14	neg	-73	neg	-18	neg	-64	neg
	10/5/2021	Neat	20	neg	121	POS	-24	neg	-68	neg	-22	neg	-67	neg
	10/12/2021	Neat	22	neg	122	POS	-21	neg	-67	neg	-17	neg	-66	neg
	10/26/2021	Neat	21	neg	123	POS	-16	neg	-69	neg	-21	neg	-55	neg
	6/19/2021	DTT	22	neg	111	POS	NT	NT	NT	NT	NT	NT	NT	NT
	8/31/2021	DTT	20	neg	115	POS	NT	NT	NT	NT	NT	NT	NT	NT
	10/5/2021	DTT	19	neg	124	POS	NT	NT	NT	NT	NT	NT	NT	NT
	10/12/2021	DTT	20	neg	119	POS	NT	NT	NT	NT	NT	NT	NT	NT
	10/26/2021	DTT	23	neg	116	POS	NT	NT	NT	NT	NT	NT	NT	NT

Note: All values are represented in median channel shift (MCS) from the negative control (i.e. patient serum MCF minus negative control MCF)
Crossmatch positive cutoff: T-cell > +39 MCS, B-cell > +70 MCS
NT, not tested
