# Supplementary material for: Daratumumab Interferes with Allogeneic Crossmatch Impacting Immunological Assessment in Solid Organ Transplantation
Source: J Clin Med. 2022 Oct 14;11(20):6059. doi: 10.3390/jcm11206059 (PMC9605360; doi:10.3390/jcm11206059)
Supplement: Supplementary file 1 [file jcm-11-06059-s001.zip › Table S4_Autologous Crossamtch.pdf]

Table S4: Autologous flow crossmatch results

	Recipient serum		T cell Crossmatch		B Cell Crossmatch	
**Crossmatch Date**	**Category**	**Date**	**Result**	**MCS**	**Result**	**MCS**
9/29/2020	Current	9/28/2020	**Negative**	34	**Negative**	-26
	Historic	6/19/2020	**Negative**	27	**Negative**	-32

MCS, Median Channel Shift

Crossmatch positive cutoff (two standard deviations above the mean of the negative control fluorescence):
T cell > +49 MCS
B cell > +71 MCS
